# Synthetic biology approaches towards the recycling of metals from the environment

**DOI:** 10.1042/BST20190837

**Published:** 2020-07-06

**Authors:** Michael J. Capeness, Louise E. Horsfall

**Affiliations:** Centre for Systems and Synthetic Biology, and the Centre for Science at Extreme Conditions, School of Biological Sciences, University of Edinburgh, Roger Land Building, Alexander Crum Brown Road, Edinburgh EH9 3FF, U.K.

**Keywords:** bioremediation, biotechnology, synthetic biology

## Abstract

Metals are a finite resource and their demand for use within existing and new technologies means metal scarcity is increasingly a global challenge. Conversely, there are areas containing such high levels of metal pollution that they are hazardous to life, and there is loss of material at every stage of the lifecycle of metals and their products. While traditional resource extraction methods are becoming less cost effective, due to a lowering quality of ore, industrial practices have begun turning to newer technologies to tap into metal resources currently locked up in contaminated land or lost in the extraction and manufacturing processes. One such technology uses biology for the remediation of metals, simultaneously extracting resources, decontaminating land, and reducing waste. Using biology for the identification and recovery of metals is considered a much ‘greener’ alternative to that of chemical methods, and this approach is about to undergo a renaissance thanks to synthetic biology. Synthetic biology couples molecular genetics with traditional engineering principles, incorporating a modular and standardised practice into the assembly of genetic parts. This has allowed the use of non-model organisms in place of the normal laboratory strains, as well as the adaption of environmentally sourced genetic material to standardised parts and practices. While synthetic biology is revolutionising the genetic capability of standard model organisms, there has been limited incursion into current practices for the biological recovery of metals from environmental sources. This mini-review will focus on some of the areas that have potential roles to play in these processes.

## Metal criticality

Both the supply of and demand for metals are at a critical point and obtaining a sufficient supply to meet this demand is a prime concern for many industries. Political, ethical, or environmental issues surrounding their extraction and trading compound the financial cost of obtaining a number of economically important metals. The recent review by the European Commission [1] has determined the current criticality of metals based on both current supply risk and economic dependency. The elements of magnesium, niobium and the platinum group metals are some of the most at risk and economically important. REE (rare earth elements) in particular are at high supply risk; though not rare on a global scale, they are not found in dense, easily exploited ores, making their extraction economically inefficient, despite an ever increasing demand for their use in current and forthcoming technologies [2]. The field of electronics for example has seen the number of different metallic elements in their production increase from 12 in the 1980's to as many as 60 in 2014 [3]. Similarly, with the transition from petrol to electric vehicles, the demand for battery grade lithium, cobalt and nickel is predicted be far in excess of current availability by 2030, and as such are also designated as critical [4].

As well as the financial and technological impetus for the recovery of metals from the environment, health and social reasons also exist. Water and land are both an expensive commodity globally and in many places they are laden with contaminant metals such as arsenic, lead and cadmium amongst others [5]. Often this contamination is a result of mining operations, but can also be due to natural occurrences in the geology, either of which result in a damaging effect to public health, and limits farming and development in those areas [6]. For all these reasons, limiting the loss of metals to the environment and addressing water and land contamination is important, and both can be achieved by shifting the paradigm of metal usage from a linear economy to that of a circular one.

**Figure 1.**
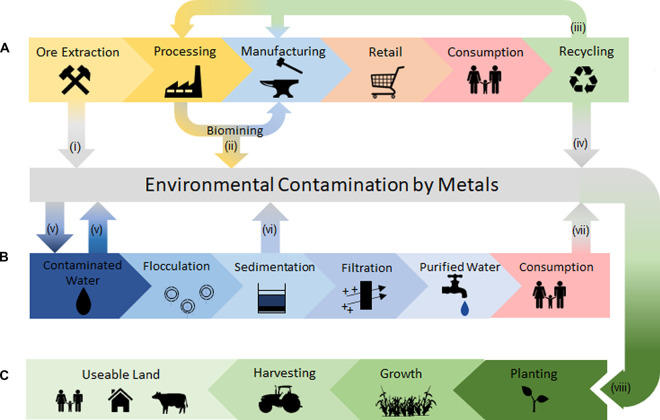
The relationship of the metal loss leading to environmental contamination with a focus on the mining industry, water purification and the decontamination of land. (**A**) The standard mining process from ore extraction to eventual consumption and recycling of metal-containing products. Metals are often lost to the environment in low grade ores where their extraction is not financially viable (**i**), similarly metals are also lost after traditional biomining has taken place (**ii**), and the resultant waste being highly acidic and requiring neutralisation. At the end of life, the metal product can either be recycled and entire the metal lifecycle once more (**iii**) or is disposed of in land fill or by incineration (**iv**) with the metals entering the environment once again. (**B**) Wastewater treatment of contaminated/non-palatable water for its eventual consumption by humans, plants and animals. Often the water contains contaminants from industrial processes or naturally occurring contaminants. During its treatment metals are removed mainly at the flocculation and sedimentation stages involving prokaryotic, eukaryotic organisms, the resultant slurry is often disposed of into the environment (**vi**). Eventual consumption of the water or its use in an industrial capacity releases it back into the environment containing contaminants (**vii**). (**C**) The cleaning of land rich in metal contaminants from industrial processes or naturally occurring geology. The planting of hyperaccumulators can remove metals from the soil (**viii**) after the growth and harvesting of the plants, freeing the land for human and agricultural use.

Currently the recovery of metals from the environment takes place through mining, water purification, and land decontamination, with dependencies therein, as illustrated in Figure 1. All three of these approaches have a biological step in which a variety of different organisms are used to recover the metals from a ‘waste’ product to either detoxify or improve cost-efficiencies.

## Biomining

After physical and chemical extraction of the high-grade ore from mined rock, the remaining debris is considered too low in metal concentration for further processing using these means (Figure 1A). By using biological treatment it is possible to extract further value from this low-grade waste ore. This waste, which is now acidic due to the previous processing treatments, can be further chemically treated with acids and cyano-compounds to solubilise the residual metals from the ore, though with little specificity for particular metals [7]. Depending on the metal contents of the ore, gold and silver can be recovered by biooxidation [8,9], and/or copper, zinc, cobalt and uranium can be captured by bioleaching [10]. In both cases, sulfuric acids are added to promote the solubility of metals ions, which are then bioabsorbed or bioaccumulated by naturally occurring, or by administered single/mixed cultures of, bacteria and archaea. During this treatment, the internal temperatures of the spent ore heap can reach up to 50°C and further acid can be added manually or can be produced by the microbes to maintain the acidic environment. Following this, the deposits are washed to recover the metal-laden microbes for down-stream processing. As well as microbes, bacterially derived extracellular polymers can also be added to promote growth of microbial cultures [11], these substances form at the interface between the spent-ore deposits and the bacteria to stimulate biofilm formation and cell-to-cell communication, to encourage an increase in metal recovery [12].

The majority of microbes used in this process are acidophilic bacteria or archaea with high heat tolerance and the ability to oxidise iron and/or sulfur containing compounds [13]. Of these microbes the *Acidithiobacillus* sp. are particularly prominent in biomining cultures, either solely or in mixed cultures including other bacteria such as *Acidiphilium* sp, *Leptospirillum* sp, and *Sulfobacillus* sp (Reviewed in Gumulya et al. [13]).

While both biomining and use of chemicals increase the recovery of metals from unspent ore, the resulting wastewater containing trace amounts of metals, acids, salts and microbes can be leached into the environment, resulting in both contaminated land and water unless contained or detoxified (Figure 1(i)).

## Wastewater treatment

While the extraction of metals from mining waste is carried out for economic reasons, the treatment of water, from both industrial application and the environment, for the removal of metals is largely for detoxification purposes instead of for commercial value. On smaller scales, and with lightly contaminated samples, filtration and ion exchange are used. However at large scale this purification often takes the form of activated sludge that is a highly dynamic community of microbes activated using aeration, and used to breakdown organic particulates, absorb metal ions, and to eventually sediment and clarify the wastewater (Figure 1B). Unlike biomining, this treatment is entirely integrated and essential to the whole water purification process. Using this activated sludge is for the targeted removal of metals and has begun to be the subject of many recent studies including for its use in the removal of chromium from wastewater [14,15] and its use in the recovery of cadmium, nickel, copper, lead and zinc [16,17].

## Contaminated soil

Unlike mining waste and wastewater, soil is less mobile and contained, making it difficult and costly for bioremediation [18]. Many physical and chemical remediation treatments for contaminated soil involve earth removal and/or replacement of the soil to remove toxic metals such as cadmium, lead, arsenic, zinc, and nickel [18], meaning that these methods are restricted in scale. However, phytoremediation using metal-accumulating plant species is being ever more considered for this purpose as it allows larger areas to be decontaminated (Figure 1C) [19]. Example accumulators include *Populus* sp. (poplar), and *Salix* sp. (willow) for cadmium removal [20], *Brassica* sp. for chromium, copper, lead and nickel removal [21], and *Pteris* sp. for the removal of arsenic [22]. After growth on contaminated soil, the entire plant or its leaves and branches are harvested, removing the metal from the site.

While all methods for the remediation of metals from the environment provide commercial and public health benefits. Using biology can be considered relatively greener and more cost-effective compared with purely chemical strategies, when taking into account the comparative cost of soil removal against how microbes and plants are inexpensive to cultivate; this is especially true as many metal accumulating species occur naturally in the areas of high metal pollution.

## Potential drawbacks

The drawback of these biological methods is the speed and efficiency at which the process is carried out. Biomining, for example, can take up to 40 days for beneficial recovery of the desirable metals, while using plants to bioremediate soil can take months to years. Also the conditions under which the biological processes take place are out the ideal range for many organisms. In addition to the high concentrations of metal ions, the environments are highly acidic/basic, are high in temperature, and osmolarity, and contain a multitude of different toxic compounds [23]. Furthermore, the biomining and sedimentation processes are highly dynamic environments, changing over the application time; so administered microbes need to be versatile and adaptable [24]. While evolution has produced organisms with natural abilities that can be utilised in metal recovery, genetic engineering would allow the creation of a robust and highly adaptable organism for the optimal removal and recovery of metals from the environment [13].

## Synthetic biology

Synthetic biology involves the application of engineering principles to molecular biology, and the adoption of the philosophy of ‘design, build, test, and learn’, allowing iterative construction and feedback loop analyses. Synthetic biology builds on existing technologies by standardising the genetic parts required to form transcriptional units and operons, and allows facile generation of libraries *in vitro* and subsequent screening for desirable phenotypes. Prior to this, biological approaches have been slow and complex, with non-standardised with incompatible parts between different methods. A number of singular and overlapping toolkits have therefore been developed for the genetic engineering of a wide variety of industrially important organisms (Table 1). These toolkits span a variety of different functions from simple expression of heterologous proteins in model systems, to improvement of the genetic capabilities of non-model organisms by way of replicating plasmids and antibiotic resistances. Currently there are limited examples of their use in the role of biological remediation and the detection of metals, as many of the toolkits are relatively new. The exception to this is that of the more established BioBrick system, for which there are examples of its implementation for the bioremediation of gold [25], cobalt and nickel [26], mercury [27] and the detection of arsenic, mercury and copper [28].

**Table 1:** Different synthetic genetic toolkits for a variety of purposes and different target organisms

System	Assembly method	Main function	Target organism/s	References
BioBricks	Type II	Expression	*E. coli*	[29]
PhytoBrick	Type IIS	Expression	Plants	[30,31]
SEVA	Type II	Replication, Expression	Various	[32]
ClosTron	Type II	Knockout	*Clostridia* sp.	[33]
CIDAR MoClo	Type IIS	Expression library	*E. coli*	[34]
EcoFlex	Type IIS	Expression, Library	*E. coli*	[35]
EasyClone	Type II	Genomic integration	*S. cerevisiae*	[36,37]
CyanoGate	Type IIS	Expression library	Cyanobacteria, Plants	[38]
JUMP	Type II, Type IIS	Expression, Universal acceptor	Various	[39]
*Chlamydomonas* MoClo	Type IIS	Expression	*Chlamydomonas reinhardtii*	[40]
pHsal series	Type II	Expression, Knockout	*Halobacterium salinarum*	[41]

Included in the table are the family of restriction enzymes used (Type II or IIS) for the assembly of the different modules into genetic circuits. Type II — restriction enzymes that cut at a specific palindromic DNA recognition site, Type II — restriction enzymes that cut a given distance away from the non-palindromic DNA recognition site. The initial assembly and generation of all vectors are carried out in standard *E. coli* cloning strains.

## Prospects for biorecovery using current strains

Model bacteria such as *E. coli* and *Bacillus*, or model yeast such as *Saccharomyces cerevisiae*, are notably absent from the field of metal biorecovery, and are often reported to be far worse at the process when compared with other non-model organisms, despite their genetic tractability [42]. The choice of chassis for the process of metal recovery is a significant one, and not to be understated. Whether to adapt a model organism, or adapt an environmental one, is of prime consideration. Likewise adoption of standardised parts to and from model and non-model organisms comes with a risk, and does not guarantee an effective outcome. Understanding the biological conditions as well as the microbial communities is also key to developing an efficient strain for the biorecovery process [43].

Though a number of toolkits are available for a wide variety of different organisms, little work has been carried out on the bacteria and archaea used in biomining or wastewater treatment. *Acidothiobacillus* sp and *Sulfolobus* sp. for example have very limited genetic tools and capabilities [13], despite them being a near-permanent presence in the biomining industry. This, however, is beginning to change; proteomic analysis of *A. ferrooxidans* has highlighted the proteins involved in both the generic stress response as well as during iron oxidation on pyrite [44]. Additionally extracellular polysaccharides, which are important in the bioleaching and oxidation processes [11], are under investigation in the hope that they encourage and enhance biofilm formation to bolster biomining efficiencies [45]. Though preliminary, these studies have generated datasets containing promising candidates for adaption, either for the native host, where applicable, or in a model heterologous host.

Using synthetic biology, as well as systems biology, the following methods can be applied to the bioremediation of metals either from the environment to improve the efficiency of metal capture and removal using standard strains, or by the adaption and domestication of organisms used in current processes.

## Detection

The first step in the recovery and removal of metals from the environment is the detection of their presence in the first place either in the ore samples, the waste water or the contaminated soil (Figure 1A–C). Currently this is achieved via a variety of chemical and physical methods, often requiring a third party, but biosensors offer a potentially greener, more cost-effective alternative alongside the opportunity to carry out the analysis *in situ*. Organisms found at sites with high metal concentrations can also be considered a repository of new genetic material for the biological detection, response mechanisms, which can be harnessed and inserted into non-heterologous hosts using synthetic biology.

The induction of a biosensor is often coupled to a visible output for easy, on the spot analysis, and GFP, chemiluminescence, or enzyme-catalyzed colour production/change have all been employed. These are engineered to be highly sensitive to low amounts (fM-mM) of single metal types with limited cross-talk [46]. While allowing the easy identification of metals in an initial analysis, biosensors can also be incorporated into more elaborate gene circuits, being used to induce metal capture pathways in an engineered organism. Current biosensors include those used for the detection of arsenic in drinking water [47], mercury and cadmium in anaerobic environments [48], gold [49], copper and silver [50], zinc, and lead [51] in soils, with some of these being *in situ* platforms for field analysis without the need for a laboratory.

As many of these metals are present in samples together, the engineering of robust, multi-elemental sensors are also desirable, which is often achieved using native regulator/sensing proteins from a wide variety of organisms. Sensing/regulators such as the CusSR system (copper), ArsR (arsenic), MerR (mercury), NikR (nickel) and the much studied Fur (iron) all exist across prokaryotes and eukaryotes alike, and are central to the cellular response to excess metal [52,53]. Adaption of these and their surrounding untranslated regions often form the starting components of the genetic circuits and can be used to initiate transcription of other genes as well as amplifying the response for more efficient metal detection using synthetic biology principles [54].

Current research has shown the ability to design complex modular synthetic gene circuits based on the BioBrick format, attuned to the specific metallic ions of lead, zinc, and cadmium with minimum cross-talk [54]. This has been achieved by specific DNA base changes in the operon encoding the promoter and ribosomal binding site modules and the inclusion of genetic amplifiers to boost the cellular response. The result is a finely tuned genetic circuit with high specificity.

## Adsorption/Chelation

**Figure 2.**
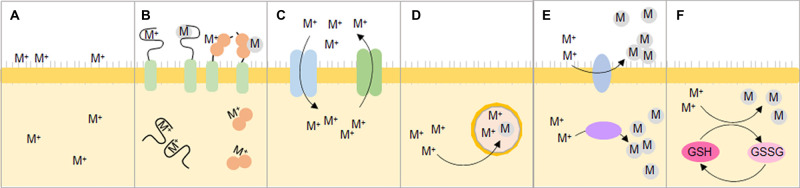
Various methods in which synthetic biology can be used to promote the capture of metals as either metal ions (M^+^) or reduced forms (M). (**A**) Absorption by an organism both on the cell surface, and intracellularly. (**B**) The addition of phytochelatins and metallothioneins for the accumulation of metals either for cell surface display, using a membrane anchor, or intracellularly. This acts not only as a platform for metal ions but for the reduction in metals. (**C**) Influx and efflux of metal ions leading to intracellular accumulation or detoxification, respectively. (**D**) The compartmentalisation of metal ions as well as their precipitation into reduced forms in nanocapsules/microcompartments. (**E**) Membrane bound, or cell-free cytochromes and hydrogenases for the reduction in metal ions to zero-valent forms either inside the cell or at the surface. (**F**) Glutathione (GSH) for the reduction in metal ions, and the regeneration of GSH from GSSG.

There are several methods by which organisms can remove and recover metal ions. One is by directly adsorbing them onto their surface, allowing the metals to be recovered at the same time as the organism when a suitable harvesting technique is employed. This is one of the primary ways in which metals can be removed during the biomining process (Figure 1A) and involves the metal ions binding to extracellular polysaccharides and/or the biofilm matrices with a range of bacteria, algae and fungi showing to do so [55,56]. While metal adsorption can occur somewhat passively, the addition of metallothioneins (MTs) and phyto-chelatins (PCs) can vastly improve this process. These allow the binding of more metals to the cell surface as well as increasing the resistance of the organisms to toxic metal ions (Figure 2A,B). Both MTs and PCs are cysteine-rich peptides that chelate metal ions via thiolate bonds and are found in wide range of different organisms [57]. The plant-derived PCs, rich in cysteine and glutamine residues, are known for their role in heavy-metal tolerance to cadmium, lead, and zinc by chelating the metal ions and keeping them away from important cellular functions either intracellular or extracellularly [58]. Varying the length and residue number of PCs has been shown to broaden their specificity to include a wider range of metal ions [59], and this has recently been applied in combination with MTs using an iterative, rational synthetic design process for the surface adsorption of copper using small cysteine-rich peptides. The targeted application is the recovery in electronic waste, and there is the possibility of expanding this further to REE and platinum containing waste [60] and can be directly applicable to the current recycling processes (Figure 1(iii)). In addition, bacteria phage have also been investigated for metal recovery, with the surface display of small peptides with a randomised design being shown to be a viable option for the chelation of the REE-containing compound lanthanum phosphate [61]. As well as simply chelating metal ions, PCs and MTs have also been used as a platform for the formation of nanoparticles for a very wide range of metals using material from their biorecovery process [62,63].

## Absorption

As well as the surface binding of desirable metals, absorption allows for intercellular accumulation providing the presence of such metals does not inhibit normal cellular physiology due to oxidative damage or mis-metalation. This is a process in which plants particularly excel at [19], and are the main candidates for the treatment of contaminated land as they are able to accrue large amounts of metal ions and the plants can be harvested to remove the offending toxins from the site (Figure 1(viii)). To achieve this, metal homeostasis as well as high oxidative stress response and intracellular chelation are essential. To this end both influx and efflux machinery work to limit the build-up of metal ions on the inside of the cell (Figure 2C). P-type ATPases are of particular importance and have been shown to play a large role within natural soil communities when in the presence of heavy metals including copper, arsenic and platinum (reviewed by Ahemad [64]). They have also recently been reported to have a role in both the cadmium and manganese response in *Lactobacillus* [65], as well as in plants [66]. Another set of metal-transporters, the ABC permeases found in Gram-positive bacteria, have been shown to promote the uptake of metal ions from the surrounding media such as soil, water, or from host cells, and in *Staphylococcus* sp. this includes, copper, cobalt, zinc, and nickel [67]. This ability can be directly applied across the whole format of biorecovery of metals (Figure 1). Similarly, the use of delftibactin, a siderophore-like non-ribosomal peptide, from *Delftia acidovorans* can scavenge gold and be re-absorbed by the bacterium for metal accumulation. Since its discovery, it has been proposed of potential use in the recovery of low-concentration gold from both ore and electronic wastes [68] and can be applied in the recycling process (Figure 1(iii)).

Other metallic ion shuttle proteins can aid in the accumulation of metals to either increase concentrations or alleviate the toxic effects that metals impose on the cell by sequestering them away from vital cellular processes. Metal-binding proteins such as CusF (copper) and SilE (silver) found across the bacterial kingdom act as ‘molecular sponges’ for metals creating a metal sink for the accumulation of metals inside the cell to negate the effects of toxic build up [69,70].

Compartmentalisation also aids in alleviating the problems associated with metal ion stress inside the cell. In plants vacuoles allow storage of toxic metals ions away from critical cell mechanisms. Similarly in microbes the diverse use of micro- and nano- compartments as well as encapsulins in prokaryotes allows for the accumulation of otherwise toxic metals ions such as iron (Figure 2D) [71]. The compartments, which are proteinaceous shells, readily assemble to form intercellular cages in which ions and proteins can be deposited, making them ideal biofactories for the production or accumulation of toxic compounds. Recently they have been adapted for the accumulation of silver and its precipitation in nanoparticle form, which otherwise un-caged would be deleterious to the host [72].

## Bioconversion

Chelation and compartmentalisation both rely on sequestration to limit the effect that the toxic ionic forms of metals have on a cell's viability, while bioconversion involves conversion of the metal ions themselves. Bioremediation occurs because of the cell's metabolism, its reduction capacity caused by the use of ATP as a terminal electron acceptor and its frequent requirement for electron shuttle-mediators such as hydrogenases, or cytochromes.

The biorecovery of metals has been proposed for a number of different organisms and particularly bacteria. Their ability for the bioremediation process, their ease of manipulation and relatively fast growth have led them to be the preferred candidates for the process of metal recapture. As such a number of species are rising to the fore, with regards to their metal accumulation and recovery abilities. These include, S*hewanella* sp. (for silver [73], manganese [74], palladium [75]), *Pseudomonas* sp. (iron, ruthenium, cobalt, platinum, palladium, lithium [76]); *Morganella* sp. (copper [77], silver [78]), *Desulfovibrio* sp. (gold [79], technetium[80], uranium [81], zinc [82] platinum and palladium [83]). Each of these organisms are at different stages with regards to their application to the industrially relevant recovery of metals and have various levels of tractability pertaining to the genetic tools and techniques that are available for them. *Pseudomonas* for example has a number of characterised induction systems and a method of recombineering [84]. While a modular plasmid toolkit, based on the BioBrick assembly method, was recently reported for use with *Shewanella* and demonstrated in the reduction in tungsten [85].

The capacity of bacteria to reduce metals is especially of note in anaerobic bacteria, and more specifically the sulfate-reducing bacteria, which rather than requiring oxygen as a terminal electron acceptor can use a variety of different metals, reducing them in the process (Figure 2E). They are of particular use in anaerobic digesters for the treatment of sewage, and for the reduction and recovery of metals either in elemental form or in complex with sulfur (Figure 1B) [86]. Sulfate metabolism is one of the main ways in which to precipitate metals but has mainly been limited to bacteria, though recently adaption of the H_2_S production pathway in *S. cerevisiae* has been shown to be an effective method, together with cell-surface display peptides for the precipitation and recovery of lead, mercury and copper [87].

Conversely, metal reduction can occur separately from metabolism and as part of the resistance mechanism to metal-ion stress, effectively removing the population of metal ions from the cytosol or surrounding medium by bio-precipitation. Of the many ways this is achieved, the most widely utilised is the glutathione (GSH) pathway. GSH reduces the metal ions, to less toxic and precipitated forms, lessening the negative impact of metal ions on critical cell processes. GSH is oxidised to GSSG during the reduction process, and then is itself reduced back to GSH by glutathione reductase, allowing it to perform its function once more (Figure 2F). This process is closely linked with the role of naturally occurring phytochelatins in the nickel and cadmium tolerance of plants, as well as general stress and immune responses in plants [88]. GSH also has a role in human copper tolerance and regulation [89], archaeal metal homeostasis [90], and bacterial metal resistance to cadmium, copper, zinc and other transition metals [91]. Recently it has even been shown to have a potential effect upon the resistance to platinum in human cells undergoing cis-platin treatment.

## Final considerations

While the process of metal chelation has been the focus of many engineering strategies there are other factors to take into account when designing strains for metal recovery, such as the temperature, pH, osmolarity and competition with the existing microbial community present in the waste streams [13]. For an effective biorecovery chassis these need to be taken into consideration and may dictate the adoption of non-model or model organisms to the task of metal recovery.

Wherever innovation is to replace existing technologies, many factors need to be taken into account that address both the limitations of the current practices as well as the initial problems of the adoptions of new technologies and the problems that they bring with them (Table 2). Though this will take place on a case-by-case basis, it is important to innovate responsibly especially when both the environment and genetically modified organisms are involved, which for many will be the limiting step. The initial adoption of synthetic biological practices will no doubt be quite an expensive undertaking involving new infrastructure and training and so will remain small scale in the first instance to provide proof of principle returns in profit. To this end, it is important that the link between industry and academia continues to strengthen to encourage the process of integration of synthetic biology in the biomining and biorecovery processes to overcome the initial hurdles if the technology is to be firmly adopted.

**Table 2:** The main positives and negatives points for the current biological methods of metal recovery and those associated for the potential adoption of synthetic biological practices

**Current Biological Methods**
*Pros:*
Current technology — Infrastructure, methodologies, technology are already in place.
Low work input — Relatively passive processes with little exogenous additions.
Low cost — The cost is outweighed by the financial return of the recovered metal.
*Cons:*
Low specificity — Many ores not being applicable (e.g. platinum, REEs).
Slow capture process — Often months are required for recovery process.
Hazardous materials — Use of acids requires neutralisation of fully spent ore before disposal.
Leaching — Both metals and acids can leach into the environment if not controlled.
**Synthetic Biological Methods**
*Pros:*
Increased metal specificity — New biological chassis increases the targeted metal remit.
Increased return — new ores and metals bring increased financial incentive.
Faster capture — Growth rate of organisms increased, recapture time of metals increased.
Adaptability — Biological chassis can be selected based on sample conditions.
*Cons:*
Future technology — New infrastructure, different materials, and skills required.
Genetically engineered organisms — Leading to the requirement for shielding from the environment.
Smaller scale — Need for containment resulting in lower turnover of samples.
Standardisation/Regulation — Requirement for the adoption of new practices across the industry.

## Perspectives

- Importance: The use of biological organisms for the recovery of metals from the environment is an important step in limiting the threat to metal criticality as well as a means for the detoxification of land and wastewater.
- Current understanding and challenges: Synthetic biology offers the genetic tools to respond to this opportunity by adapting current organisms or more model-organisms to improve this recovery process. Though at the small scale currently, its adoption to the industrial level, will require both a political and social acceptance of genetically modified organism.
- Future directions: Synthetic biology will continue to spread to an even wider number of organisms and help in the replacement of technologies currently carried out by chemical methods.

## Open Access

Open access for this article was enabled by the participation of University of Edinburgh in an all-inclusive *Read & Publish* pilot with Portland Press and the Biochemical Society under a transformative agreement with JISC.

## Abbreviations

fM: Femtomolar
mM: Millimolar
MoClo: Modular Cloning
MTs: Metallothioneins
PCs: Phytochelatins
